# Knowledge, attitude and preventive practice of cutaneous leishmaniasis in Sodo district, Ethiopia

**DOI:** 10.1038/s41598-025-30623-z

**Published:** 2025-12-02

**Authors:** Lina Gazu, Zerish Zethu Nkosi, Nigatu Kebede

**Affiliations:** 1 Animal Health and Zoonotic Disease Research Unit, Aklilu Lemma Institute of Pathobiology, Addis Ababa University, Addis Ababa, Ethiopia; 2 Department of Health Studies , University of South Africa , Pretoria , South Africa

**Keywords:** Knowledge, Attitude, Practice, Cutaneous leishmaniasis, Sodo district, Ethiopia, Diseases, Health care

## Abstract

Cutaneous Leishmaniasis (CL) is a neglected tropical disease endemic in Ethiopia, causing significant morbidity, disfigurement, and social stigma. Despite its public health importance, community awareness and preventive behaviours, particularly in Sodo District, remain inadequately characterised. This study aimed to assess knowledge, attitudes, and practices (KAP) related to CL among residents of Sodo District, Southern Ethiopia, with emphasis on transmission and vector biology, and risk perceptions. In this cross-sectional descriptive study, data were collected via structured, pre-tested questionnaires from 423 systematically selected households in six communities of Sodo District between January and April 2018. Cumulative KAP scores were dichotomised at the mean (noted to be normally distributed) to define satisfactory or unsatisfactory knowledge, favourable or unfavourable attitude, and good or poor preventive practice. We used descriptive analysis to summarise data and logistic regression to identify associations between higher KAP scores and sociodemographic variables. Associations among KAP domains were assessed using the Phi coefficient. The questionnaire was pretested for clarity; ethical approval was obtained, and verbal informed consent was documented for all participants. Among 423 participants, 61.9% demonstrated satisfactory knowledge, 53.4% had a favourable attitude, and 50.4% reported good preventive practices. Notably, only 51.1% recognized CL lesions from images, while over half were unaware of the causative agent and transmission routes. Awareness of vector behaviour was limited; fewer than half were knowledgeable about sandfly biting and breeding sites. Most participants acknowledged CL as a serious community health problem with disfiguring consequences if untreated. Use of preventive measures such as bed nets was moderate (68.1% ownership; 59.5% consistent use), but repellent use was low (30.3%). Personal contact with someone affected by CL significantly predicted better KAP. Phi coefficients indicated statistically significant positive associations among KAP domains, highlighting their interrelationships. Community awareness and prevention practices in Sodo District show substantial gaps, especially regarding vector biology and transmission. These findings underscore the urgent need for targeted, evidence-based health education interventions. Such interventions should include community-based awareness programmes, school outreach initiatives, and integration with local health extension services to enhance knowledge, promote positive attitudes, and improve preventive behaviours. Tailoring education to the local context and vulnerable groups will be essential for increasing uptake and ultimately reducing CL transmission in endemic areas like Sodo District.

**Supplementary Information:**

The online version contains supplementary material available at 10.1038/s41598-025-30623-z.

## Introduction

Cutaneous Leishmaniasis (CL) is a parasitic disease transmitted by the bite of infected female sandflies^1^. It primarily affects the skin, beginning as erythematous papules that progress to nodules and eventually ulcerated, crusted lesions at the bite site due to parasite replication in the dermis. Although CL is not fatal, it results in permanent scarring, physical disability, and significant social stigma, particularly affecting vulnerable groups such as children and women^2^. Globally, over one million new CL cases occur annually, with major endemic hotspots in countries including Afghanistan, Iran, Brazil, and Peru^3^. Recently, environmental changes, host immune factors, treatment failures, and emerging drug resistance have contributed to the re-emergence of CL in many endemic regions^4^.

In Ethiopia, CL poses a significant public health challenge, with an estimated annual burden ranging between 20,000 and 50,000 new cases annually^5^. Meta-analyses report a pooled prevalence of approximately 19.0% (95% CI: 14.0–24.0%). However, the high heterogeneity of studies (I^2^ = 99.9%) indicates substantial reginal variation, warranting cautious interpretation and further local investigations^2^. Retrospective data from treatment centres in the Amhara region show variable CL prevalence ranging from 2.3 to 49 cases per 10,000 outpatients, while molecular studies confirm its widespread endemicity driven by environmental and behavioural factors^6^. In a newly identified endemic area in South Ethiopia, the prevalence of active CL was estimated at 2.5%. Risk factors include the presence of animal dung in household compounds and spending time outdoors in areas inhabited by hyraxes, potential reservoir hosts for the parasite^7^. Emerging evidence also indicates CL outbreaks at lower elevations, such as the Somali lowlands (~ 500 m)^8^. This contrasts with the historically endemic highland areas (1,400-2,000 m) especially in Amhara, Tigray, Oromia, and the Southern Nations, Nationalities, and People’s Region (SNNPR), now reorganised as the South Ethiopia Regional State^9^. Data from Kutaber district, Ethiopia, also indicate high local prevalence, but values vary with sociodemographic factors and prevention practices^10^. These findings highlight the heterogeneous epidemiology of CL across Ethiopia’s diverse regions and the critical need for ongoing molecular surveillance, tailored public health strategies, and more precise regional prevalence assessments to guide control efforts.

*Leishmania aethiopica* is the predominant causative species of CL in Ethiopia and is characterised by diverse clinical manifestations, including localized, diffuse, and mucocutaneous forms^11^. Diffuse CL often fails to heal spontaneously and can relapse after treatment, while mucocutaneous CL may involve the nasal or oral mucosa, leading to eating and breathing difficulties^12^. Lesions caused by *L. aethiopica* commonly occur on the face, especially among children and young adults, and tend to present with atypical and more severe clinical features than those caused by other *Leishmania* species^13^. The disfiguring effects of CL cause significant psychological distress, including anxiety and depression, which in turn reduces quality of life and economic productivity in affected communities^14^. Locally, CL is known by various names, for example, “Kunchir” in Gojam, “Finchoftu” in northern Shoa, and “Bolbo” in Ochollo, reflecting its deep social and cultural roots^15^. Despite these burdens, Ethiopia currently lacks a national vector control programme or human vaccines for CL^9^. The Ethiopian Federal Ministry of Health has prioritised leishmaniasis control by focusing on vector management, community education, and enhanced surveillance^16^.

Knowledge, attitudes, and practices (KAP) related to CL in Ethiopian communities remain poorly characterised, impeding effective awareness and prevention campaigns. Several localised studies reveal widespread misconceptions about disease transmission, low uptake of preventive practices, and stigma-associated barriers to care. For instance, in Ethiopia, communities in Kutaber^17^ and Wolaita zone^18^ demonstrate moderate awareness but inconsistent protective practices, while stigmatising attitudes in areas like Ochollo^19^ lead to underreporting and delays in treatment seeking. These findings underscore the urgent need for localised KAP assessments to tailor health education and control efforts appropriately.

This study systematically assesses the KAP regarding CL among residents of Sodo District, Southern Ethiopia, a high-incidence area within the former Southern Nations, Nationalities, and Peoples’ Region (now South Ethiopia Regional State). The district’s epidemiological and sociodemographic profile shares key characteristics with other CL-endemic zones across Ethiopia, making it a useful context for understanding community perceptions and behaviours related to CL. The findings will inform targeted public health strategies and awareness campaigns adapted to the local context and contribute to broader efforts to reduce the burden of CL in Ethiopia.

## Methods

### Description of the study area

**Fig. 1:**
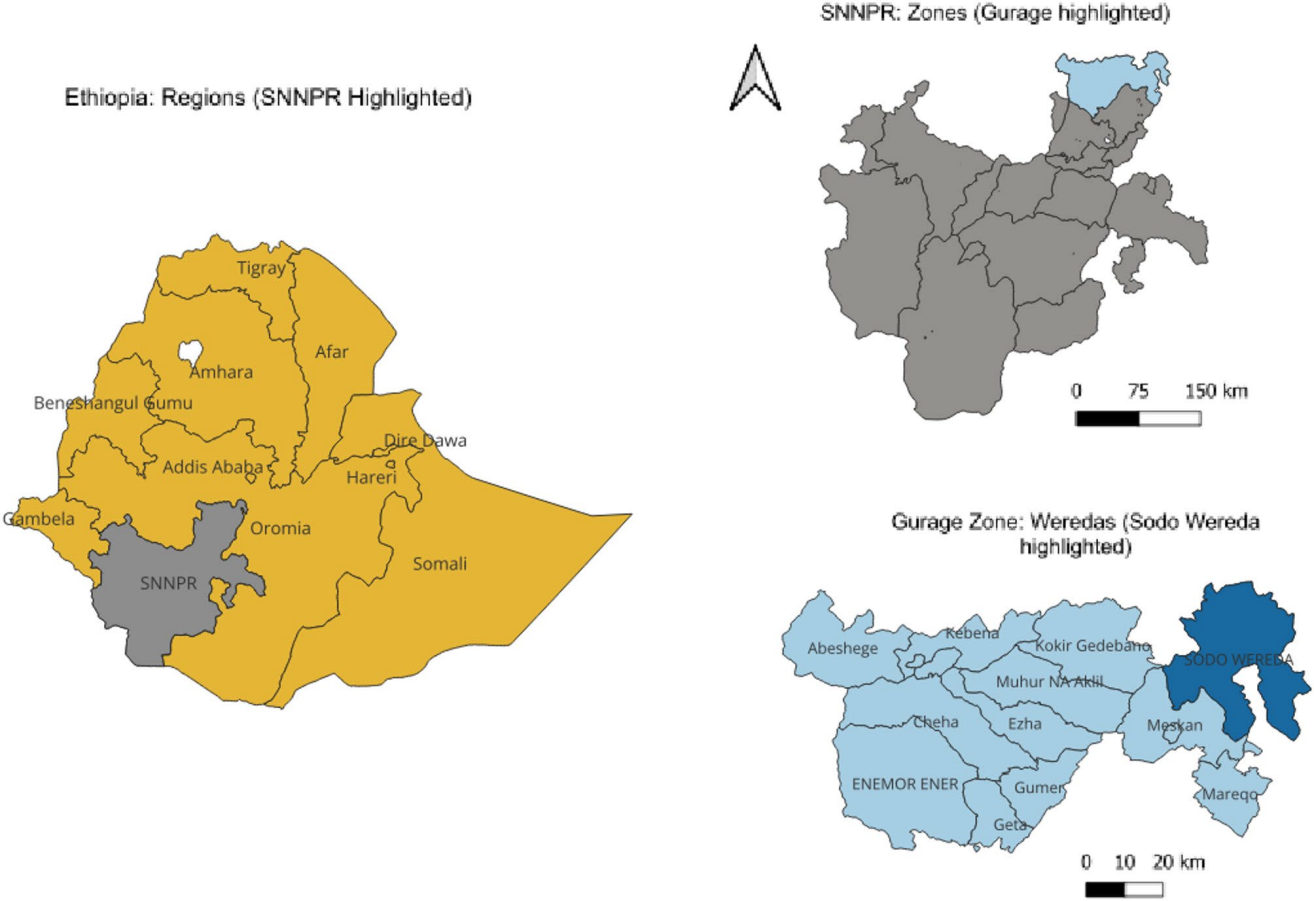
Map of study area (Sodo District, South Ethiopia Regional State). SNNPR (currently, there are two additional regions, namely Sidama and Southwest Ethiopia, which were created after the survey was conducted). The base layer dataset included shapefiles obtained from open AFRICA (https://africaopendata.org/dataset/ethiopia-shapfiles*)* which is licensed under a Creative Commons Attribution licence. The map was created using QGIS version 3.20.1.

The research was carried out in Sodo District. Sodo District is situated in Gurage Zone of Southern Nations, Nationalities, and Peoples’ Regional (SNNPR) state, now reorganized as the South Ethiopia Regional State (Fig. 1). The geographical location of the district is between 8° 09’ to 8° 45’ North latitude and 38° 37’ to 38° 71’ East longitude. It is located approximately 103 km south of Addis Ababa. The district has an area of 88,553.3 hectares.

The district has a total population of 175,725 people, with 89,619 (51.0%) women and 90% of the population residing in rural regions. Within the district, there are eight health centres, 55 health posts, two private clinics, and three pharmacies. Based on case reports obtained from the Sodo District Health Office, supplemented by consultations with local health workers and traditional healers (unpublished administrative data), six kebeles, namely Kela Zuria, Kola Nurena, Michael Semero, Adazer, Beka, and Genete Mariam, were selected as the highest CL reported areas. The number of households (2632 in total) in each of these kebele is shown in Table 1. The total number of households for each kebele was obtained from the District Administration Office.

**Table 1:** Total number of households and population in selected Kebeles (*N* = 2632).

Kebele*	No. of households	Population
Kela Zuria	333	2028
Kola Nurena	362	2389
Michael Semero	475	3055
Adazer	610	3477
Beka	299	1678
Genete Mariam	553	2912

*Kebele is the smallest administrative unit in Ethiopia.

### Design, sample, and sampling methods

A cross-sectional survey was developed to evaluate the KAP of the community regarding CL.

For determining the sample size, 50% of the communities were assumed to be knowledgeable about CL with a 2% margin of error and 95% confidence interval. The following single population proportion formula was employed to get the definite sample size with a 10% non-response rate:

$$n=\frac{{{\text{Z}}^{\text{2}}}_{{{\text{1}}/{\text{2 }}*}}p\left( {{\text{1}} - p} \right)}{{d^{\text{2}}}}$$

Where: *d* = margin of error (5%), Z_1/2_ = 1.96 for 95% confidence, *p* = estimated prevalence (50%), *q* = 1 − *p*.

Adding a 10% non-response rate, the required sample size was 423 households.

Formal power calculations were not conducted; however, the sample size of 423 meets recommended criteria of a minimum of 10–20 outcome events per predictor variable ensuring statistical power (> 80%) for logistic regression. Finite population correction (FPC) was not applied due to the large population size, which would minimally affect sample size.

The total sample was divided according to the proportion of households in each kebele using:

$${n_i}=\frac{{{\text{N}}_{\text{i}}} \times n}{N}$$

where *n*_*i*_ = sample size for kebele *i*, *N*_*i*_= number of households in kebele *i*, and *N* = total households in the six kebeles.

Therefore, 54,58,76,98,48 and 89 questionnaires were administered in Kela Zuria, Kola Nurena, Michael Semero, Adazer, Beka, and Genete Mariam, respectively. Households in each kebele were systematically selected from updated household lists provided by district authorities. For each kebele, the sampling interval (k) was calculated by dividing the total number of households listed in that kebele by the number of households to be surveyed. Every k^th^ household was then selected until the required sample size was reached. Within selected households, the head of household or, if absent, an available adult member was invited to participate. Households that could not be contacted after three visits were considered non-responsive and replaced. Because the survey targeted household heads or, in their absence, available adult members, younger populations were underrepresented, which may influence the generalizability of age-related findings.

### Data collection

A standardized questionnaire (Supplementary Text S1) was prepared and administered to each participant who agreed to be part of the study. The survey tool was translated into Amharic language keeping its original meaning.

The questionnaire was administered by nine trained health extension workers with a diploma or bachelor’s degree. The data collectors received a one-day training before commencing data collection. Interviewers read the questions verbatim but allowed participants to respond freely without prompting or guiding answers to minimize bias. The household heads or the oldest available adult were interviewed.

### Data quality assurance

The questionnaire was pre-tested with 30 individuals outside the study population to evaluate clarity, cultural relevance, and understanding. Throughout data collection, the principal investigator checked completed questionnaires daily for completeness and consistency.

### Ethical considerations

The research was approved by the Ethics Committee of the University of South Africa (UNISA) Department of Health Studies (Reference number: REC-012714-039/HSHDC/457/2015). The SNNPR health bureau, Gurage zone administration, and Sodo Health office all issued approval letters. Verbal informed consent was obtained from all participants. All procedures were conducted in accordance with relevant guidelines and regulations.

### Inclusion and exclusion criteria

Participants comprised all consenting individuals residing in the selected six kebeles. Individuals living outside these kebeles were excluded.

### Data analysis

The survey questionnaire comprised two sections: sociodemographic characteristics and KAP questionnaires. The KAP section included 8 items assessing knowledge, 6 items assessing attitude, and 7 items assessing preventive practice: these focused on disease cause, treatment, transmission, signs and symptoms, the vector, and preventive measures. All participants were shown images of a typical CL lesions, sourced from peer-reviewed clinical study conducted in Ethiopia^13^ and further validated by an expert from the All Africa Leprosy, Tuberculosis and Rehabilitation Training (ALERT) centre. All participants, regardless of prior awareness of CL, were shown the lesion photo to standardize the assessment of lesion recognition.

To assess knowledge and recognition of the sandfly vector, participants were also presented with an image of the sandfly vector (Supplementary Figure S1). This image was also validated by entomologist from Addis Ababa University, Aklilu Lemma Institute of Pathobiology (ALIPB), to ensure relevance and accuracy in the local Ethiopian context. This visual approach helped to standardize lesion and vector recognition among participants while minimizing potential biases.

Each item was scored as 1 for a correct (or favorable) response, and 0 for incorrect or ‘don’t know’ responses. Scores for each domain were summed to yield composite scores. These continuous scores were then dichotomized using the sample mean as the cut-off, classifying participants scoring above the mean as having satisfactory knowledge, favorable attitude, or good practice, and those below as unsatisfactory, unfavorable, or poor. No fixed minimum score was applied because knowledge and attitudes towards CL were expected to vary widely in this community, including among individuals with no prior awareness of the disease. Using the sample mean allowed relative comparison within the study population and ensured consistency with prior KAP studies in similar endemic settings. However, this approach may have misclassified some participants with low absolute scores as having ‘satisfactory’ knowledge or attitudes, a limitation acknowledged in the interpretation of results.

A binary logistic regression analysis (both bivariate and multivariate) was conducted to identify sociodemographic predictors of KAP outcomes, reporting crude and adjusted odds ratios with 95% confidence intervals. Predictor variables included age, sex, education, occupation, knowing someone infected with CL (yes/no), and place of birth (within the district or another district).

Variable selection for the multivariate model was guided by Akaike’s Information Criterion (AIC), an established method that balances model fit and complexity by penalizing over-parameterization to select the optimal subset of predictors. All candidate variables from the bivariate analysis were initially considered, and stepwise selection based on AIC minimization was applied to identify the final model. Interaction terms were assessed but excluded as they did not improve model fit. Collinearity diagnostics were performed to avoid multicollinearity among predictors. The final model, with 96% cumulative weight, included main-effect variables only.

Phi coefficients were calculated to assess associations among the dichotomized KAP domains, as this statistic is appropriate for binary variables. All analyses were conducted in R version 3.02, compatible with necessary packages.

## Results

### Socio-demographic characteristics

Of 423 participants, 219 (51.8%) were male and 204 (48.2%) were female. Age was categorized into continuous 10-year intervals beginning at 18 years (e.g., 18.0–24.4.0.4, 24.5–34.4, etc.) to ensure mutually exclusive and non-overlapping groups. The largest age groups were 34.5–44.4 years (35.5%), 24.5–34.4 years (25.3%), and 44.5–54.4 years (19.4%). Most participants were Orthodox Christians (92.4%) which was the dominant religion in Sodo District. Regarding educational attainment, 216 (51.1%) could not read or write, while 28.1% were able to read and write but did not receive formal education. The main occupation was farmer (51.1%); the remainder were housewives, traders, or other roles (Table 2).

The average family size was 5.76. Only a small proportion of the study participants (*n* = 81, 19.1%) came from other places while the majority (*n* = 342, 80.9%) were born in the district (Table 2).

**Table 2:** Demographic characteristics of the respondents (*N* = 423).

Characteristics	Frequency	Percent
Sex		
Male	219	51.8
Female	204	48.2
Age		
18.0–24.4	24	5.7
24.5–34.4	107	25.3
34.5–44.4	150	35.5
44.5–54.4	82	19.4
Over 54.5	60	14.2
Religion		
Orthodox	391	92.4
Protestant	29	6.9
Muslim	3	0.7
Educational level		
Illiterate (cannot read & write)	216	51.1
Can read and write only	119	28.1
Primary education (1–6 grade)	63	14.9
Secondary education (8–12 grade) and above	25	5.9
Occupation		
Farmer	216	51.1
Housewives	188	44.4
Trader and other	19	4.5
Place of birth		
Same district	342	80.9
Another place	81	19.1

### Knowledge of the community about CL

To assess recognition, participants were presented with images of CL cases. Among the 423 participants, 216 (51.1%) correctly identified the condition, whereas 350 (82.7%) reported having previously heard of CL (Table 3). All subsequent KAP questions use the full sample (*n* = 423) as the denominator unless otherwise specified. Most participants cited family, friends, and neighbours as their source of information about CL (82.7%), while others reported learning about CL from colleagues (67.5%) or through personal experience (9.0%).

When asked about the cause of CL, only a small proportion of participants correctly identified a germ as the cause, while more than half reported not knowing. Plaque-like and pus-containing lesions were the most frequently recognized signs and symptoms. The nose (*n* = 316, 74.5%), cheeks (*n* = 305, 71.9%), forehead (*n* = 116, 27.4%), and ears (*n* = 102, 24.1%) were most commonly cited as lesion sites (Table 3).

Understanding of disease transmission was limited. Only 12.3% identified the sandfly as the vector responsible for CL, and confusion with other vectors, such as mosquitoes, was common. To further gauge knowledge of the sandfly vector, participants were shown a picture of sandfly prior to questioning about the biting and blood-feeding behaviour. As a result, just under half of respondents (49.6%) demonstrated awareness of the sandfly as a biting and blood-sucking vector. Among those who identified vector breeding sites, the most frequently mentioned were dirty places (51.5%), water ponds (45.9%), and garbage collection sites (26.2%). Only a small proportion mentioned crevices in the house (4.3%), thatched roofs (4.0%), or cattle sheds (4.7%).

**Table 3:** Knowledge of CL among study participants (*N* = 423).

Variables (multiple responses possible)		Frequency	Percentage
Heard about CL	Yes	350	82.7
	No	73	17.2
Major cause of CL	Do not know	226	53.4
	Germ	52	12.3
	Other	46	10.9
	Drinking unclean water	44	10.4
Sign and symptoms	Fever	56	13.2
	Plaque	268	63.4
	Skin rash	56	13.2
	Pus containing	206	48.7
	Papule	55	13.2
	Do not know	92	21.7
Disease transmission	Mosquito bite	30	7.1
	Contact with patient	24	5.7
	Sandfly bite	52	12.3
	Do not know	252	59.4
Biting and blood-sucking behaviour of the vector	Aware	210	49.6
	Not aware	198	46.8
Knowledge (overall)	Satisfactory	263	61.9
	Unsatisfactory	161	38.1

**Fig. 2:**
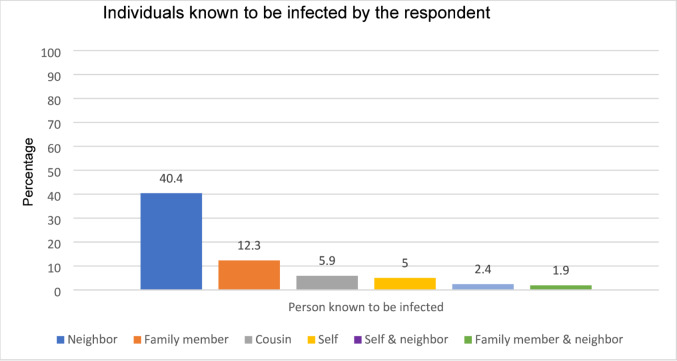
Relationship of persons known to be infected with CL and respondent.

Most (*n* = 301, 71.2%) of the respondents knew someone infected with CL in their area. According to the respondents, neighbours were most commonly identified as individuals known to be infected with CL (40.4%), followed by family members (12.3%), cousins (5.9%), and the respondents themselves (5.0%) (Fig. 2).

### Factors associated with knowledge of CL

In bivariate analysis, female sex (*p* = 0.001), primary education (*p* = 0.01), being a housewife (*p* = 0.001), and being born outside the Sodo district (*p* = 0.03) were significantly associated with higher knowledge of CL. Not knowing someone with CL was negatively associated with knowledge (*p* < 0.001). However, after adjustment, only primary education and knowing someone with CL remained significant predictors. Primary education remained strongly associated with higher knowledge (AOR = 3.88, 95% CI: 1.73–8.70, *p* = 0.001). Notably, those unaware of anyone with CL had markedly lower odds of satisfactory knowledge (AOR = 0.16, 95% CI: 0.10–0.27, *p* = 0.001). Other factors, including age, sex, occupation, and place of birth, did not show significant associations after adjustment (Table 4).

**Table 4:** Bivariate and multivariable odds ratios and corresponding p-values for comparison of variables with knowledge of CL (*N* = 423).

Variables	COR (95% CI)	AOR (95%CI)	*P*-value (AOR)
Sex			
Male	1	1	
Female	2.90 (1.92–4.37)	2.00 (0.64–6.23)	0.233
Age			
18.0–24.4	1	1	
24.5–34.4	1.35 (0.54–3.39)	0.95 (0.34–2.67)	0.922
34.5–44.4	1.01 (0.41–2.45)	0.88 (0.32–2.42)	0.804
44.5–54.4	0.99 (0.39–2.52)	1.08 (0.37–3.16)	0.888
Over 54.5	0.53 (0.20–1.39)	0.66 (0.22–2.03)	0.464
Educational level			
Illiterate	1	1	
Can read and write only	0.49 (0.31–0.77)	0.84 (0.47–1.49)	0.541
Primary	2.52 (1.24–5.11)	3.88 (1.73–8.70)	0.001
Secondary and above	0.49 (0.21–1.13)	0.61 (0.24–1.58)	0.31
Occupation			
Farmer	1	1	
Trader	1.68 (0.64–4.44)	1.04 (0.30–3.65)	0.952
Housewife	2.94 (1.90–4.50)	1.53 (0.48–4.89)	0.475
Place of birth			
Same district	1	1	
Another place	1.84 (1.08–3.14)	0.75 (0.37–1.52)	0.423
Know a person with CL infection			
Yes	1	1	
No	0.15 (0.09–0.25)	0.16 (0.10–0.27)	0.001

### Attitude towards CL in Sodo district

Overall, 226 participants (53.4%) demonstrated a favourable attitude (Table 5). Most respondents (61.7%) believed that CL can be treated. The majority recognized the seriousness of CL as a community health problem (64.5%) and acknowledged the risk of disfiguring outcomes if untreated (62.4%). Willingness to participate in control programmes was high, with 77.5% of participants expressing readiness to engage.

**Table 5:** Attitude of the Sodo community towards CL (*N* = 423).

Variables (Multiple responses are possible)		Frequency	Percentage
CL can be treated	Yes	261	61.7
	No	70	16.5
	Do not know	85	20.1
Seriousness of CL in the community	Very serious	273	64.5
	Ordinary	31	7.3
	Not very serious	46	10.9
	Do not know	66	15.6
	Other	6	1.4
Outcome of CL if untreated	Death	28	6.6
	Disfiguring	264	62.4
	Self-cure	1	0.2
	Both (death & disfigure)	67	15.8
	Do not know	54	12.8
	Other	8	1.9
Feel well informed about CL	Yes	167	39.5
	No	248	58.6
	Do not know	7	1.6
Willing to participated in CL control programmes	Yes	328	77.5
	No	82	19.4
	Do not know	12	2.8
Preferred choice of medication for CL	Modern medicine	142	33.6
	Herbal medicine	251	59.3
	Do not know	28	6.6
Attitude (overall)	Favourable	226	53.4
	Unfavourable	197	46.6

**Fig. 3:**
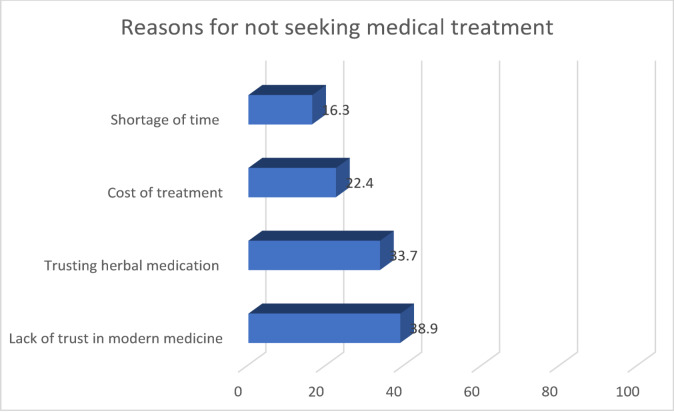
Reasons for not seeking medical treatment.

Over half (59.3%) preferred traditional herbal medicine as a treatment option. Only about one third favoured modern medical care. The main reasons cited for avoiding modern care included distrust in conventional medication (*n* = 165, 38.9%), confidence in herbal remedies (*n* = 143, 33.7%), high cost of modern treatments (*n* = 95, 22.4%), and limited time to seek medical care due to demanding farm work (*n* = 69, 16.3%) (Fig. 3).

Major barriers identified by participants to controlling CL in their community included lack of awareness (*n* = 171, 40.3%), limited financial resources (*n* = 114, 26.9%), and reliance on herbal medicine (*n* = 99, 23.3%). The absence of awareness programs was further highlighted by the fact that 248 (58.6%) respondents felt they were not well informed about CL. Nearly half (*n* = 203, 47.9%) attributed their lack of knowledge to insufficient educational opportunities and inadequate information sources in their locality.

### Factors affecting community’s attitude towards CL

In bivariate analysis (Table 6), female sex (*p* = 0.02), age groups 24.5–44.4 (*p* = 0.048) and 34.5–44.4 (*p* = 0.02) years and being a housewife (*p* = 0.03) were significantly associated with a favourable attitude towards CL. Not knowing someone with CL was associated with markedly lower odds of a favourable attitude (*p* = 0.001). After adjusting for confounders, the age group 34.5–44.4 years and being able to read and write remained significant predictors of a favourable attitude. Participants unaware of anyone with CL continued to show substantially lower odds of a favourable attitude (AOR = 0.22, 95% CI: 0.14–0.36, *p* = 0.001). Other variables, including sex, occupation, and place of birth, did not show significant associations after adjustment.

**Table 6:** Comparison of socio-demographic variables with the attitude to CL (*N* = 423).

Variables	COR (95% CI)	AOR (95%CI)	*P*-value (AOR)
Sex			
Male	1	1	
Female	1.58 (1.08–2.33)	1.28 (0.455–3.588)	0.644
Age			
18.0–24.4	1	1	
24.5–34.4	2.55 (1.01–6.48)	2.57 (0.930–7.093)	0.068
34.5–44.4	3.00 (1.21–7.45)	3.58 (1.299–9.890)	0.014
44.5–54.4	2.21 (0.85–5.72)	2.59 (0.905–7.400.905.400)	0.075
Over 54.5	1.43 (0.53–3.85)	2.36 (0.726–7.305)	0.152
Educational level			
Illiterate	1	1	
Can read and write only	1.16 (0.74–1.82)	1.93 (1.10–3.40)	0.022
Primary	1.61 (0.92–2.89)	1.90 (0.98–3.66)	0.058
Secondary and above	1.50 (0.65–3.49)	2.37 (0.92–6.08)	0.073
Occupation			
Farmer	1	1	
Trader	0.95 (0.37–2.43)	0.84 (0.23–2.74)	0.776
Housewife	1.56 (1.05–2.31)	1.35 (0.47–3.85)	0.58
Place of birth			
Same district	1	1	
Another place	0.54 (0.33–0.90)	1.61 (0.85–3.06)	0.144
Know a person with CL infection			
Yes	1	1	
No	0.21 (0.13–0.34)	0.22 (0.14–0.36)	0.001

### Prevention practice of Sodo community regarding CL

Overall, prevention practice scores were evenly distributed around the mean, indicating moderate variability in preventive behaviours among participants. About half (50.4%) demonstrated good prevention practice, while 46.9% showed poor practice. When asked how CL was treated, for 301 participants who know someone infected with CL, application of herbal remedies (*n* = 150, 49.8%) and the use of specific drugs prescribed by physician at health centre (*n* = 90, 30.0%) were more frequently mentioned (Table 7).

About half (*n* = 235, 55.6%) of participants used preventive measures. Most (68.1%) households owned a bed net, but only 59.5% of those used them consistently at night. Looking into the number of bed nets, of those who use bed nets, 105 (24.8%) and 69 (16.3%) had a maximum of two or three bed nets in the house, respectively. Far fewer used other preventive measures (only 30.3% used repellents). Any type of insecticide had never been sprayed in 210 (49.5%) of the participants’ houses. Of the 170 (40.0%) insecticide sprayed houses, the purpose was for protection against malaria. Few (4.5%) reported sleeping outside, a recognized risk factor. Preferred time of work at peak temperature was daytime (*n* = 148, 35.0%) and both at daytime and night (39.0%) (Table 7).

**Table 7:** Treatment and preventive practice of the Sodo community against CL (*N* = 423).

Variables (multiple responses)		Frequency	Percentage
How was CL treated*	Herbal	150	49.8
	Praying	15	5
	Drugs given at health centre	90	30
	Not treated	6	2
	Holy water	13	4.4
	Do not know	27	8.9
Use of preventable measures	Yes	235	55.6
	No	130	30.7
	Do not know	44	10.4
Having bed net in the house	Yes	288	68.1
	No	124	29.3
	Do not know	11	2.6
Always using bed net when sleeping^$^	Yes	171	59.5
	No	92	31.9
	Do not know	25	8.6
Use of repellents	Yes	128	30.3
	No	244	57.7
	Do not know	51	12
Sleeping outside or spending time outside	Yes	19	4.5
	No	392	92.7
Work time preference at high T^o^	Daytime	148	35
	Night	37	8.7
	Both	165	39
	Very early morning	44	10.4
Practice (overall)	Good	213	50.4
	Poor	201	46.9

*n = 301 number of individuals who know someone with CL and ^$^n = 288 number of individuals who have bed net in the house.

### Factors affecting the preventive practice of CL

In bivariate analysis (Table 8), female participants (*p* = 0.001), housewives (*p* = 0.001), and those born outside Sodo (*p* = 0.003) had higher odds of good prevention practice, while participants aged over 54.5 years (*p* = 0.02) and those able to read and write only (*p* = 0.03) had lower odds. Not knowing someone with CL (*p* = 0.001) was also strongly associated with lower prevention practice. After adjusting for confounders, only knowing someone with CL remained a significant independent predictor, with those who do not know anyone with CL being significantly less likely to engage in preventive measures (AOR = 0.32, 95% CI: 0.20–0.51, *p* < 0.001). Other factors, including sex, age, education, occupation, and place of birth, did not retain significance (Table 8).

**Table 8:** Socio-demographic characters and preventive practice of participants (*N* = 423).

Variables	COR (95% CI)	AOR (95%CI)	*P*-value (AOR)
Sex			
Male	1	1	
Female	2.36 (1.59–3.48)	0.84 (0.31–2.29)	0.734
Age			
18.0–24.4	1	1	
24.5–34.4	0.93 (0.37–2.31)	0.77 (0.29–2.02)	0.593
34.5–44.4	0.50 (0.21–1.21)	0.45 (0.17–1.18)	0.106
44.5–54.4	0.70 (0.27–1.78)	0.70 (0.26–1.91)	0.48
Over 54.5	0.32 (0.12–0.86)	0.36 (0.12–1.05)	0.062
Educational level			
Illiterate	1	1	
Can read and write only	0.61 (0.38–0.95)	0.85 (0.49–1.48)	0.566
Primary	1.56 (0.87–2.77)	1.80 (0.95–3.44)	0.072
Secondary and above	0.70 (0.31–1.63)	0.78 (0.31–1.95)	0.592
Occupation			
Farmer	1	1	
Trader	1.36 (0.53–3.49)	1.35 (0.42–4.28)	0.62
Housewife	2.55 (1.71–3.90)	2.14 (0.77–5.97)	0.143
Place of birth			
Same district	1	1	
Another place	2.15 (1.30–3.57)	1.34 (0.72–2.48)	0.351
Knowing someone with CL			
Yes	1	1	
No	0.30 (0.19–0.47)	0.32 (0.20–0.51)	0.001

### Overview of overall KAP of CL

Comparing overall KAP (Table 9), among participants with satisfactory knowledge (*n* = 263), 65.8% (173/263) demonstrated good prevention practices, and 64.6% (170/263) had a favourable attitude towards CL. Similarly, among those with a favourable attitude (*n* = 226), 66.4% (150/226) engaged in good prevention practices.

**Table 9:** Comparison of overall KAP (*N* = 423).

Knowledge	Attitude	Unfavourable	Favourable	Total (*n*, %)
Unsatisfactory		104 (64.6%)	56 (35.4%)	160 (100%)
Satisfactory		93 (35.4%)	170 (64.6%)	263 (100%)
	Total	197 (46.6%)	226 (53.4%)	423 (100%)

Knowledge	Practice	Poor	Good	Total (*n*, %)
Unsatisfactory		121 (75.6%)	40 (24.4%)	161 (100%)
Satisfactory		89 (33.8%)	173 (65.8%)	263 (100%)
	Total	210 (49.3%)	213 (50.7%)	423 (100%)

Attitude	Practice	Poor	Good	Total (*n*, %)
Unfavourable		134 (68.0%)	63 (32.0%)	197 (100%)
Favourable		76 (33.6%)	150 (66.4%)	226 (100%)

Table 10 presents the Phi coefficients along with their interpretations and significance levels. All associations were statistically significant (*p* < 0.001), indicating moderate positive relationships among the KAP components with phi values ranging from 0.31 to 0.57.

**Table 10:** Phi coefficients measuring associations between KAP domains among participants (*N* = 423).

Variables	Phi Coefficient (φ)	*p*-value
Knowledge - Practice	0.572	< 0.001
Knowledge - Attitude	0.386	< 0.001
Attitude - Practice	0.306	< 0.001

## Discussion

In this study, 51.1% of participants correctly identified cases of CL, and 82.7% had heard about the disease, figures which are notably higher than those reported in other Ethiopian areas such as Ochello in Gamo Gofa Zone^19^, as well as international settings including India^20^, Paraguay^21^, and Southwestern Iran^22^. This relative community awareness likely reflects the endemic status of CL in the Sodo district and its long history in Ethiopia^15,23,24^. Despite such familiarity, significant knowledge gaps persist; over half of respondents did not know the causative agent (53.4%) or transmission routes (59.4%), similar to studies in Ethiopia^6,17,19^. Comparatively, higher transmission knowledge levels have been recorded in Ecuador^25^ and Iran^22^, which may reflect differences in disease endemicity, health system engagement, or the extent of community awareness initiatives in those contexts.

Knowledge of the sandfly vector which is critical for transmission was limited, with only 49.6% aware of its biting and blood-feeding behaviour, and confusion between sandflies and mosquitoes was common. Low awareness of the vector has been similarly documented in the Tigray region of Ethiopia^26^, Pakistan^3^, and southwestern Iran^22^. Sandflies are nocturnal and prefer microhabitats with organic matter and moisture for breeding^27,28^. Our data also revealed poor knowledge of vector breeding sites, a deficit that hinders adoption of effective preventive measures. This deficit is critical as a case-control study from Nepal demonstrated that insecticide-treated bed nets significantly reduce visceral leishmaniasis risk, a finding potentially relevant to CL as well^27^. Behavioural models such as the Health Belief Model illustrate how perceived susceptibility and severity, mediated by knowledge, influence health behaviours^29,30^.

The community’s preference for traditional herbal treatments (59.3%) over biomedical therapies reflects limited access to medical services and entrenched cultural beliefs. Such reliance on indigenous remedies has similarly been observed in Colombia and French Guiana^31,32^. In Ethiopia, few health facilities are equipped for CL diagnosis and treatment, driving patients towards traditional healers or home remedies^33^. The stigma and psychological impact of disfiguring CL lesions represent a recognized barrier to care and add complexity to treatment uptake and community engagement^14^. Stigmatization, especially in facial lesions among young and female individuals, may discourage early treatment seeking and lead to social isolation, as observed in other endemic regions^34^. Psychosocial support and community education addressing stigma should be integrated into CL control programmes.

In terms of preventive practices, 171 (59.5%) of participants reported using bed nets when sleeping. This low proportion of bed net use is similar to that reported in communities in Pakistan^3^, and Paraguay^35^. By contrast, higher bed net use was seen among 85.0% of post kala azar dermal leishmaniasis patients in India^36^. Evidence suggests that insecticide-treated nets (ITNs) used by at least half of community members can confer community-wide protection against malaria^37^. Given that sandflies are largely nocturnal and can bite both indoors and outdoors, ITNs may help reduce exposure to CL vectors, although evidence of their effectiveness in preventing leishmaniasis transmission remains inconclusive^38,39^. However, proper utilisation of bed net and bed net availability per household was insufficient in this study. While Ethiopia’s malaria prevention program targets 100% household coverage with two ITNs per household in malaria-endemic regions^16^, the geographic overlap between malaria and leishmaniasis is incomplete^40^. This underscores the need for a dedicated national leishmaniasis control strategy. Moreover, 57.7% of participants did not use repellents, although repellents are recommended to reduce insect bites by making hosts less attractive to vectors^41^. Public health messaging should place greater emphasis on the role of repellents and clarify their relevance not only for mosquitoes but also for sandflies.

Our findings indicate that CL is widely recognized as a community-level health issue. Most participants identified neighbours as the individuals they knew to be infected, and a large majority cited family, friends, and neighbours as their primary sources of information about CL (82.7%). This broad social exposure suggests that KAP towards CL are shaped by community interactions and shared experiences rather than being confined to individual households. Such pervasive community-level recognition highlights the importance of designing prevention and health education interventions that engage not only families but also wider social networks, leveraging trusted community relationships to enhance outreach, reduce stigma, and promote effective preventive behaviours.

Education level emerged as the strongest independent determinant of KAP towards CL in this study, underscoring the role of formal education in shaping disease understanding and preventive behaviours. Participants with higher education showed significantly higher awareness and preventive practices, reflecting broader access to health information, literacy, and engagement with formal health systems. This finding aligns with previous studies in Ethiopia and other endemic settings that highlight education as a key driver of leishmaniasis awareness and preventive practices^42^. While sex and occupation showed some associations with KAP in unadjusted analyses, these differences were attenuated after controlling for education, suggesting that observed gender gaps may partly reflect differences in educational attainment and access to information. Nonetheless, qualitative evidence indicates that gendered norms and social roles can still shape prevention behaviours in subtle ways, warranting further exploration of behaviours^34,42,43^. Overall, improving access to health education, particularly for populations with limited formal schooling, remains essential to enhancing community knowledge and prevention of CL.

A notable feature of our sample is that the majority of participants were above 24.4 years of age, the result of selecting household heads or the next available adult for interviews. While this approach aligns with household-based survey convention, it may underrepresent younger community members who, according to national and regional epidemiological data, bear a substantial portion of the CL burden and face particular risks related to transmission and stigma^10,13,33^. Furthermore, most participants had educational attainment below the primary level, reflecting structural barriers to health literacy and preventive practice. Given that youth often serve as community change agents, future research should directly assess KAP among younger age groups. Engaging and educating youth, both in schools and through peer-led interventions, may catalyse broader improvements in community awareness, attitudes, and adoption of effective preventive behaviours^44^. In this study, community knowledge about CL was found to be shared mostly through informal communication and social networks; empowering youth with accurate information could help correct misconceptions and promote informed prevention practices.

Analysis of the relationships between KAP domains utilised Phi coefficients, which revealed statistically significant moderate positive associations among all KAP components (all *p* < 0.001). Specifically, knowledge was most strongly associated with practice and attitude, while the attitude-practice association was also positive but weaker. These findings supported the view that higher knowledge is linked to more favourable attitudes and better preventive behaviours. However, despite these associations, overall uptake of key prevention practices, such as consistent use of bed nets and repellents, remained suboptimal. As documented in neglected tropical disease literature, knowledge alone did not always translate into action, especially when practical, social, or cultural barriers persisted^43^. Therefore, enhanced community engagement and evidence-based education remain essential for sustained improvements in CL prevention and control. Building on the Health Belief Model (HBM), which has been demonstrated to be effective in promoting preventive behaviours by addressing perceived susceptibility, severity, benefits, barriers, cues to action, and self-efficacy in CL endemic populations^42,45^. These theories emphasize constructs like self-efficacy, social norms, and behavioural intentions, providing a more comprehensive understanding of factors influencing preventive behaviours in endemic communities, as supported by applications in neglected tropical disease and vector-borne disease contexts^43,45^.

Notably, the study did not evaluate awareness or perceptions related to animal reservoirs such as the rock hyrax, a key zoonotic host for *Leishmania aethiopica* in Ethiopia^13^. This omission limits our understanding of zoonotic transmission risks and represents a missed opportunity for designing integrated control strategies. Future research should adopt a One Health perspective to explore the community’s knowledge of animal reservoirs and their role in disease ecology. Future research should evaluate interventions for vector identification and avoidance, community implementation challenges, and the effectiveness and safety of locally used herbal remedies.

Policy implications are clear: culturally sensitive and locally tailored health education programmes are essential to bridge knowledge gaps and promote preventive measures. Integrating trusted community actors, such as traditional healers, schools, media, and community health workers, can enhance message credibility and program effectiveness^16^. Health messages should focus on improving community understanding of sandfly behaviour, including identification of breeding and resting sites and awareness of nocturnal biting times, to encourage consistent adoption of effective protection strategies. Emphasizing practical, context-specific measures and differentiated approaches targeting individuals with lower educational attainment, youth, and outdoor workers can help address distinct exposure patterns, comprehension levels, and risk perceptions.

### Limitation of the study

This study’s findings are subject to several limitations. First, purposive sampling of high incidence kebeles and reliance on self-reported data may introduce selection, reporting, recall, and social desirability bias, potentially inflating estimates of awareness and preventive practice. The cross-sectional design precludes causal inference. The use of the sample mean as a cutoff for categorizing KAP levels may have led to misclassification of some participants with relatively low absolute scores as having “satisfactory” knowledge or “favourable” attitudes, particularly given the inclusion of individuals with no prior awareness of CL. This relative scoring approach reflects population-specific performance but limits comparability with externally validated benchmarks. The age profile of participants, predominantly adult household heads, may also underrepresent youth perspectives, which are relevant for community-level transmission and behaviour change. Finally, although validated images helped standardize CL and vector recognition, static photos cannot fully capture clinical or behavioural diversity, which may affect the accuracy of participants’ practical diagnostic skills.

## Conclusion

This study found that while a majority of participants possessed satisfactory general knowledge about CL, significant deficits remain, particularly regarding understanding of disease causation and the sandfly vector, which are vital for effective prevention. Even with moderate recognition of CL, knowledge of transmission was limited, increasing the risk of ongoing transmission in this endemic district. Although participants demonstrated a generally favourable attitude toward prevention and willingness to engage in control activities, actual preventive practice, particularly use of personal protective measures such as repellents and consistent use of bed nets, remained suboptimal. These gaps highlight the need for targeted, evidence-based interventions to improve knowledge and translate positive attitudes into consistent risk-reducing behaviours.

### Recommendations

- To address gaps, there is an urgent need for focused, evidence-based health education programmes tailored to the local context, incorporating community outreach, school-based education, and collaboration with local health extension services to enhance knowledge, foster positive attitudes, and encourage effective preventive behaviours.
- Priority should be given to strengthening education efforts that clearly explain the causes and modes of transmission of CL, with a special emphasis on the biology and behaviour of the sandfly vector, including typical breeding and resting sites, as well as peak biting times.
- Health promotion activities must be delivered via trusted and culturally relevant channels, including traditional healers, health workers, schools, and local media platforms, to effectively address misconceptions and reduce stigma surrounding the disease.
- It is essential to improve access to and acceptance of modern biomedical treatments for CL, ensuring that diagnostic and therapeutic services are affordable, available, and culturally appropriate within endemic areas.
- Promotion of consistent use of personal protective measures, such as insecticide-treated bed nets and repellents, should be intensified. Where feasible, CL prevention messages can be integrated with existing malaria control programmes while recognizing and adapting to the distinctive ecology and transmission patterns of leishmaniasis.
- Education and intervention strategies should be tailored for populations with lower literacy and consider age, gender, and occupation to maximise reach and equity in health outcomes.
- Lastly, fostering community participation and ownership of CL prevention and control activities is critical. Engaging local knowledge systems while simultaneously correcting prevalent misconceptions will help sustain behavioural changes and strengthen the impact of control programmes.

## Supplementary Information

Below is the link to the electronic supplementary material.

Supplementary Material 1

Supplementary Material 2

## Data Availability

The datasets supporting the conclusions of this study are included in the supplementary materials. Supplementary Data S1 contains the anonymized survey responses used for analysis (Survey_Data.xlsx).
