# Supplementary material for: Knowledge, attitude and preventive practice of cutaneous leishmaniasis in Sodo district, Ethiopia
Source: Sci Rep. 2025 Dec 2;16:1092. doi: 10.1038/s41598-025-30623-z (PMC12789511; doi:10.1038/s41598-025-30623-z)
Supplement: Supplementary file 1 — Supplementary Material 1 [file 41598_2025_30623_MOESM1_ESM.docx]

**Supplementary Information**

This file contains the supplementary materials referenced in the manuscript:

**Supplementary Table S1. KAP Survey Questionnaire (English version)**

**Legend:**
Structured questionnaire used to assess knowledge, attitudes, and practices regarding CL among residents of Sodo District, Ethiopia. The tool includes demographic information, 8 items on knowledge, 6 items on attitude, and 7 items on preventive practices.

**Survey objective:** To explore CL- related knowledge, attitudes and practice of persons bitten by suspected rabid animals

*Check one:* Date: / /

Baseline data collection; or site: Follow-up data collection code:

*Information to read to respondent:*

We'd like to learn more about your CL knowledge, attitudes, and practices. We intend to identify your requirements and the most effective manner to deliver information to you, as well as any barriers to getting medical care. CL control will be improved with the information you supply. Your responses will be kept private and will not be shared with anybody. Your name will not appear on the questionnaire or be recorded in any other way. Your participation in the interview is entirely voluntary, and you may terminate it at any time.

Thank you for your assistance.

**Interviewer:** Place an X in the box of the selected answer(s).

Do not read responses unless the directions

indicate.

# DEMOGRAPHIC INFORMATION

- 1. How old are you? Years**.**
     1. 🞎18.0-24.4 3. 🞎34.5- 44.4
     2. 🞎24.5–34.4 4. 🞎44.5-54.4

5. 🞎Over 54.5

- 1. What is your gender? (Record by observing)
     1. 🞎Male 2. 🞎Female
  2. What is the highest level of education you have completed?
     1. 🞎 illiterate (Can’t read and write)
     2. 🞎Can read and write (only)
     3. 🞎Primary education (1-6 grade)
     4. 🞎Secondary and above
     5. 🞎Other:
  3. Duration of stay at residence (In years)
  4. Family size
  5. Place of birth: 1. 🞎Sodo 2. 🞎 Another place
  6. If you come from another place; Place of origin year of arrival
  7. Occupation
     1. 🞎Farmer
     2. 🞎Housewives
     3. 🞎Trader
     4. 🞎Business
     5. 🞎Government employee
     6. 🞎Other (specify):
  8. . Religion
     1. 🞎Orthodox 5. 🞎Pagan
     2. 🞎Muslim 6. 🞎Other (specify):
     3. 🞎Protestant
     4. 🞎Catholic
  9. Do you know someone infected with CL or have you been exposed before?
     1. 🞎Yes 2. 🞎No

3. 🞎I do not remember

# KNOWLEDGE OF CL

1. Ask the respondent if they could name the disease after showing a picture of CL manifestation
   1. 🞎Able to identify as CL 2. 🞎Unable to identify
2. Have you heard about Cutaneous Leishmaniasis (Chewi)?
   1. 🞎Yes 2. 🞎No
3. If your answer is ‘yes” to the question above, how did you hear about CL? (Please choose the three most effective sources.)
   1. 🞎Newspapers and magazines 2.🞎TV

3. 🞎Billboards 4.🞎Health workers

5. 🞎Religious leaders 6. 🞎 Radio

7. 🞎Brochures, posters and other printed materials 8.🞎Teachers

9. 🞎Family, friends, neighbors and colleagues

10. 🞎 Other (please explain):

1. What is the cause of CL?
   1. 🞎spiritual or hereditary
   2. 🞎Virus
   3. 🞎Leishmania
   4. 🞎Germ
   5. 🞎I do not know
   6. 🞎other:
2. What are the symptom/signs of CL?
   1. 🞎Fever with chills 2. 🞎Plaque

3. 🞎Skin rash 4. 🞎 Papule

5. Ulcer 6. 🞎I do not know

7. 🞎other:

1. How CL transmit from one person to another?
   1. 🞎 Contact with patient
   2. 🞎 Sandfly bite
   3. 🞎 Mosquito bite
   4. 🞎 I do not know
   5. 🞎 other:
2. Where in the body are lesions/scars of CL Located?
   1. 🞎 Forehead 2. 🞎 Cheeks

3. 🞎 Arm 4. 🞎Leg

5. 🞎 Mixed 6. 🞎 Ear

7. 🞎Nose 8. 🞎I do not know

9. 🞎 other:

1. Ask the respondent if they know about the biting and blood sucking behavior after showing picture of sand fly.
   1. 🞎Yes, I know 2. 🞎 No, I don’t know
2. Where do you think is the breeding place of the fly?
   1. 🞎 Dirty place 4. 🞎 Damp and dark places
   2. 🞎 Cervices in the house 5. 🞎 water ponds
   3. 🞎Thatched roof 6. 🞎 Garbage collection sites
3. 🞎Cattle sheds
4. 🞎 do not know
5. 🞎 Others:

# THE ATTITUDES TOWARDS CL

1. Do you think CL can be treated?
   1. 🞎 Yes 2. 🞎 No

3. 🞎 I have no idea

1. What do you think the outcome of CL if not treated?
   1. 🞎Death 2. 🞎Disfiguring

3. 🞎Self-cure 4. 🞎 I have no idea

5. 🞎 Other:

1. What is yours preferred drug of choice for treatment of CL?
   1. 🞎Specific medicine
   2. 🞎Indigenous medicine
   3. 🞎 Do not know
   4. 🞎other:
2. If you would not go to the health facility, what is the reason? (Please check all that apply.)
   1. 🞎 Not sure where to go
   2. 🞎Cost
   3. 🞎 Difficulties with transportation/distance to clinic
   4. 🞎 Do not trust medical workers
   5. 🞎 Do not like attitude of medical workers
   6. 🞎 Cannot leave work
   7. 🞎 Don’t want to find out that something is wrong
   8. 🞎 preference to use herbal medication
   9. 🞎 Other (please explain):
3. Do you feel well informed about CL?

3. 🞎 Yes 2. 🞎 No

1. If your answer to the above question is “no” why is that?
   1. 🞎You not giving it attention
   2. 🞎You never had the chance to learn about rabies
   3. 🞎There is inadequate source of information in your locality
   4. 🞎other:
2. Are you willing to participated in CL control activities?
   1. 🞎 Yes 2. 🞎 No (reason: )
3. . How serious a problem do you think CL is in your locality? (Check one.)
   1. 🞎 Very serious 2. 🞎 Ordinary

3. 🞎 Not very serious 4. 🞎 I don’t know

1. What do you think are the major constraints to control CL?
   1. 🞎 Insufficient budget 3. 🞎 Religious taboo
   2. 🞎Trained professionals 4. 🞎 Lack of appropriate legislation

5. 🞎 Lack of awareness 6. 🞎 Lack of proper coordination

7. 🞎 I do not know

8. 🞎Others:

# PRACTICES REGARDING CL

1. What did you apply, or what was applied to a person you know was/is infected with CL? (Check all that are mentioned.)

| a. Herbal remedies | 1. 🞎Yes | 2. 🞎No |
| --- | --- | --- |
| b. Home rest without medicine | 1. 🞎Yes | 2. 🞎No |
| c. Praying | 1. 🞎Yes | 2. 🞎No |
| d. Specific drugs given by health centre | 1. 🞎Yes | 2. 🞎No |
| e. Holy water | 1. 🞎Yes | 2. 🞎No |
| f. Do not know |  |  |

g. 🞎Other:

1. Have you used any preventive measure against CL?
   1. 🞎 Yes 2. 🞎 No

3. 🞎I have no idea

1. Do you have bed net in the house?
   1. 🞎 Yes (how many: )
   2. 🞎 No
2. Do you have custom of spending time outside in the night or sleeping outdoors?
   1. 🞎Yes
   2. 🞎 No
3. Do you use bed nets when sleeping?
   1. 🞎 Yes
   2. 🞎 No
4. When is your Work time preference when the temperature is high?
   1. 🞎Day time
   2. 🞎Night
   3. 🞎Bot
5. Do you use repellents?
   1. 🞎Yes
   2. 🞎 No
6. Has your house ever been sprayed?
   1. 🞎 yes
      - How often;
      - Reason:
   2. 🞎 No
   3. 🞎 I don’t remember

Thank you very much for participating in our survey

## Supplementary Figure S1. Sand Fly Image for Vector Recognition


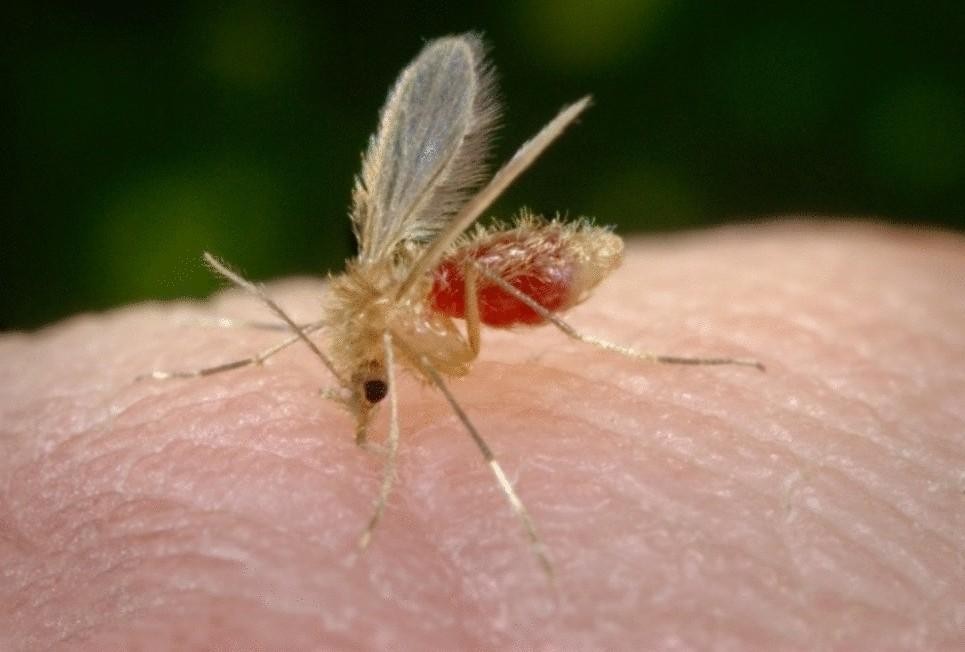
**Legend:**
Reference image of a sand fly used to assist respondents in recognizing the biting and blood-feeding behavior relevant to CL transmission.
Source: CDC Public Health Image Library (PHIL #10275), Centers for Disease Control and Prevention, USA..

**Supplementary Data S1. Survey data**

File: Survey_Data.xlsx

Dataset containing anonymized survey responses supporting the findings reported in this study.
